# Endocrine Disruptor Impacts on Fish From Chile: The Influence of Wastewaters

**DOI:** 10.3389/fendo.2021.611281

**Published:** 2021-03-25

**Authors:** Ricardo O. Barra, Gustavo Chiang, Maria Fernanda Saavedra, Rodrigo Orrego, Mark R. Servos, L. Mark Hewitt, Mark E. McMaster, Paulina Bahamonde, Felipe Tucca, Kelly R. Munkittrick

**Affiliations:** ^1^ Faculty of Environmental Sciences and EULA-Chile Centre, University of Concepción, Concepción, Chile; ^2^ Faculty of Life Sciences, Universidad Andres Bello, Santiago, Chile; ^3^ Natural Science Institute Alexander von Humboldt, Faculty of Marine Sciences and Biological Resources, University of Antofagasta, Antofagasta, Chile; ^4^ University of Waterloo, Waterloo, ON, Canada; ^5^ Water Science and Technology, Environment and Climate Change Canada, Burlington, ON, Canada; ^6^ Laboratory of Aquatic Environmental Research, Centro de Estudios Avanzados-HUB Ambiental UPLA, Universidad de Playa Ancha, Valparaíso, Chile; ^7^ Núcleo Milenio INVASAL, Concepción, Chile; ^8^ Instituto Tecnológico del Salmón (INTESAL), Puerto Montt, Chile; ^9^ Biological Sciences, University of Calgary, Calgary, AB, Canada

**Keywords:** pulp mill effluents, urban treated discharges, Biobio River, Itata River, Native fish Chile

## Abstract

Industrial wastewaters and urban discharges contain complex mixtures of chemicals capable of impacting reproductive performance in freshwater fish, called endocrine-disrupting compounds (EDCs). In Chile, the issue was highlighted by our group beginning over 15 years ago, by analyzing the impacts of pulp and paper mill effluents (PPME) in the Biobio, Itata, and Cruces River basins. All of the rivers studied are important freshwater ecosystems located in the Mediterranean region of Central Chile, each with a unique fish biodiversity. Sequentially, we developed a strategy based on laboratory assays, semicontrolled-field experiments (e.g., caging) and wild fish population assessments to explore the issue of reproductive impacts on both introduced and native fish in Chile. The integration of watershed, field, and laboratory studies was effective at understanding the endocrine responses in Chilean freshwater systems. The studies demonstrated that regardless of the type of treatment, pulp mill effluents can contain compounds capable of impacting endocrine systems. Urban wastewater treatment plant effluents (WWTP) were also investigated using the same integrated strategy. Although not directly compared, PPME and WWTP effluent seem to cause similar estrogenic effects in fish after waterborne exposure, with differing intensities. This body of work underscores the urgent need for further studies on the basic biology of Chilean native fish species, and an improved understanding on reproductive development and variability across Chilean ecosystems. The lack of knowledge of the ontogeny of Chilean fish, especially maturation and sexual development, with an emphasis on associated habitats and landscapes, are impediment factors for their conservation and protection against the threat of EDCs. The assessment of effects on native species in the receiving environment is critical for supporting and designing protective regulations and remediation strategies, and for conserving the unique Chilean fish biodiversity.

## Introduction

Endocrine disruption (ED) has become a serious environmental threat across the world (1), and there is global concern for the potential endocrine impacts of individual chemicals and mixtures. Evidence coming from developing countries has been limited, especially related impacts to the unique biodiversity in aquatic ecosystems. There are no current data available relating chemical pollution to biodiversity. Therefore, a robust documentation of the impacts of the discharge of pollutants by industrial and urban wastewaters on reproductive outcomes of fish inhabiting rivers in these jurisdictions is needed (2).

Traditionally, in Latin America, the assessment of pollution is mainly based on chemical analysis of point source discharges, and little data is available on the effects that these discharges cause on the associated fauna in the receiving environment. There is a lack of understanding therefore, on the actual impacts of wastewater discharges on the aquatic organisms, despite numerous records of pollution occurring in the region (3).

In this paper, we review the primary point source complex mixtures that are released into surface waters that exert endocrine disrupting effects in fish in Chilean freshwater aquatic ecosystems. The diversity of these potentially bioactive, endocrine-disrupting compounds (EDCs) represents a huge analytical challenge for addressing their occurrence in surface waters and effluents. Challenges include the absence of methods capable of identifying EDCs in complex mixtures, the latency period between exposure to EDCs during sensitive life stages and the manifestation of adverse outcomes, and regulatory requirements for endocrine disruptors. Unlike other types of toxicity such as dermal irritation or carcinogenicity, there are a broad array of ED endpoints that increase the complexity of the issue. Furthermore, some endocrine responsive endpoints can be altered through a variety of non-endocrine pathways (4).

The history of our journey began in the early 2000s, when new pulp mills were constructed in Chile and began releasing effluents into rivers such as the Biobio in South Central Chile, adding to existing industrial and agricultural discharges. Our initial priority was concern about potential dioxin emissions from the pulp mills (5), which attracted considerable regulatory and public attention. Scientific evidence at that time also showed reproductive effects in fish inhabiting receiving environments downstream of pulp mill discharges in Canada and Sweden (6, 7). Although dioxin contamination was a concern for potential human health impacts, it became clear that other chemicals were involved in environmental impacts as reproductive alterations in fish were present even after secondary treatment (8). To compound the issue, a number of Chilean native fish are considered threatened, and reproductive strategies and behavior of these small-bodied fishes was largely unknown. Similarly, wastewater treatment plant effluents (WWTPEs) had been demonstrated to also have EDC effects which then raised the question for the potential of these discharges having reproductive impacts on the Chilean receiving environment. Chile had started a process to enhance municipal wastewater treatment plants with secondary treatment in the early 2000s. Based on the Canadian experience, researchers in Chile developed an approach using a triad of methods including laboratory bioassays, semi-controlled field exposure experiments, and field monitoring studies (9).

### Chilean Freshwater Fish Biodiversity

**Figure 1:**
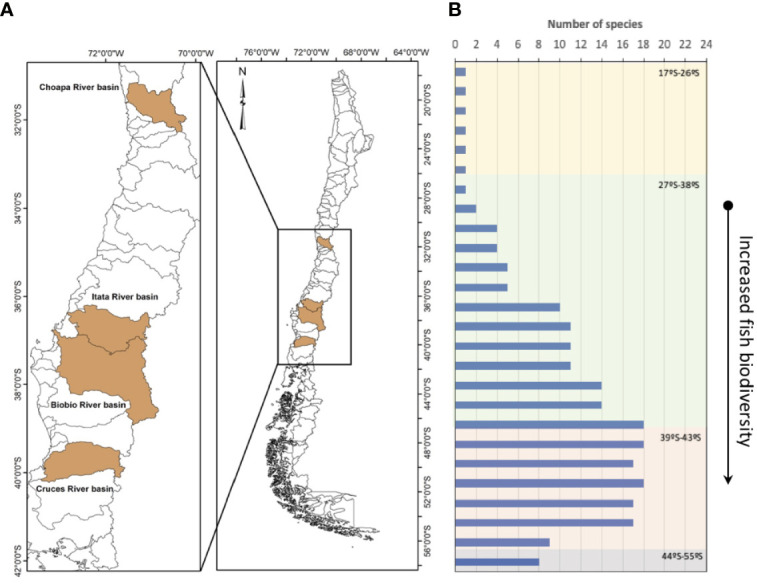
Location of the rivers where endocrine disruption effects have been described in Chile **(A)** and the latitudinal fish biodiversity reported by the Ministry of the Environment (10) (MoE) 2018 **(B)**.

In landscape terms, continental Chile has two defining structural characteristics: a latitudinal gradient that goes from 18 to 56 degrees south latitude, and an altitudinal gradient, which goes from ocean trenches 8,000 m deep to mountains at 7,000 m altitude (**Figure 1**). This makes Chile a highly heterogeneous country in relation to the environmental conditions that allows it to sustain its biological diversity (**Figure 1B**). Thus, the terrestrial part of the country is separated to the east of the continent by the Andes, to the north by the Atacama and Tarapaca Deserts, and to the south and west, by the vast Pacific Ocean. These characteristics, in conjunction with the country’s geological history, provided the conditions for the existence of unique species for the Chilean territory, almost as an island in the continent (10). This situation is clearly reflected in the low number of freshwater fish species in Chile, represented by 11 families, 17 genera, and almost 50 species, with 40% of the species classified as endangered (11), and several being restricted to few watersheds. The threatened status of native fish species is noteworthy, considering that about 80% of Chilean fish species are endemic, with a high retention of basal characteristics, low diversity, general small body sizes and adaptation to rivers with high slopes and fluctuating flow (12–15). This results in a fish fauna of high biogeographic and conservation value. There are however, important gaps in knowledge about their distribution and, above all, basic biology of these species (11).

**Figure 2:**
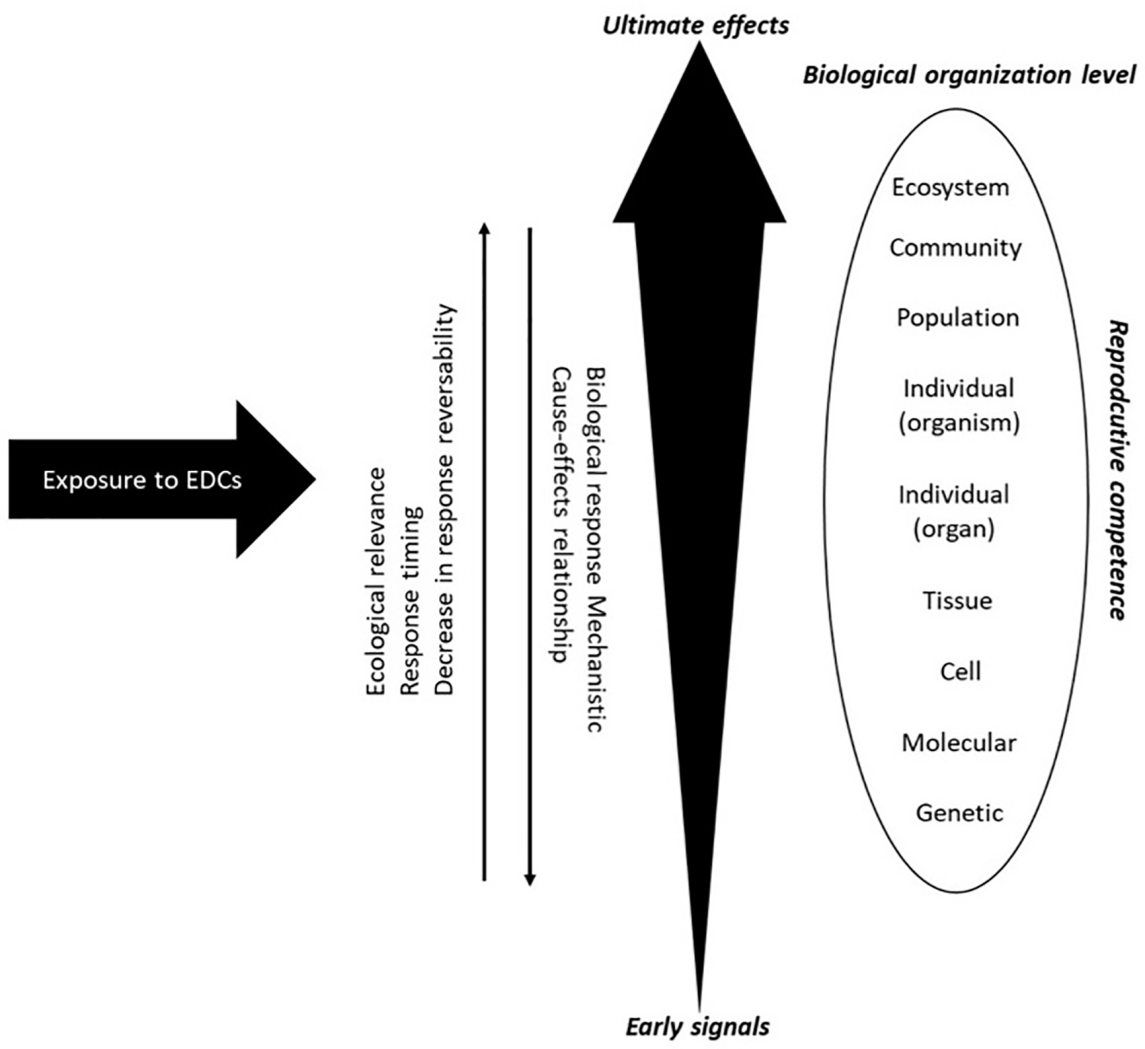
Schematic representation of stress responses within a biological system (Modified from 21, 22).

Currently, pollution of freshwater aquatic systems is one of the most important, but sometimes overlooked threats to biodiversity (16, 17). The nature of pollutants vary in different basins, depending on the industrial, agricultural, and urban activities that occur within in each watershed. Investigating the consequences of the wide occurrence of toxic substances in natural environments is therefore a major challenge. Aquatic ecosystems, which are particularly at risk, present a diverse assembly of interacting species, each with its own characteristics and habitats. This biological diversity is a great challenge for the evaluation of ecotoxicological effects of EDCs, since each species may respond differently to the same compounds or levels of exposure to toxic substances. To understand and attempt to predict the possible consequences of these substances on species requires knowledge of the biological and ecological factors that determine their sensitivities (18). Thus, among these factors, the selection of the spatio-temporal scale of the analysis has great relevance in assessing effects (19). Measures based on community changes are useful to establish the condition of the ecosystem and reveal deteriorations, but are limited in their utility to establish the causes of any changes. The temporality of these responses (on scales of generations and years) is a factor that sometimes prevents the implementation of criteria for the prevention of deleterious effects, and is a fundamental consideration for the restoration of aquatic systems (19). Conversely, parameters at the individual level (biochemical and physiological variables), are highly useful due to the shorter time response to establish linkages to the causes of the observed changes (20), despite having less ecological relevance (**Figure 2**).

In any field study, information on baselines and reference areas are critical for providing understanding and an indication of potential impact. The evaluation of natural variability in metabolic factors of growth and reproduction, in relatively clean systems, are needed to be able to interpret the effects of EDCs and estimate the relevance of these changes at higher levels of biological organization. Thus, an effective approach for early warning signs of environmental risk needs to balance the two extremes of a slow but highly relevant ecological response and rapid molecular initiating events that are mechanistically informative but difficult to link to the effects of concern. Effects measured at the individual level could explain alterations in the structure of populations and community in the aquatic environment, which may be due to direct effects of an EDC in those species, as well as the interaction with other species and habitats (19). Effects measured at the level of the individual or population may also be desirable for environmental monitoring as effects can be detected prior to the loss of a species.

This review analyzes the evidence to date for reproductive responses in fish downstream of pulp mill and urban point source discharges in Chile and recommends how the protection of fish biodiversity in rivers could be managed and sustained.

## Evidence of Pulp Mill Effluent Impacts on Non-Native Fish

A pollution gradient of suspected EDCs such as polychlorinated biphenyls (PCBs), polycyclic aromatic hydrocarbons (PAHs), pesticides (e.g. lindane), polychlorinated dibenzo-*p*-dioxins (PCDDs) and polychlorinated dibenzofurans (PCDFs) were reported in the Biobio River basin (5, 23–25) (see **Figure 1** for the Biobio river location) impacting overall water quality from the Andean Mountains (relatively unpolluted) to the river outlet (severely polluted). Previous studies provided evidence that water and sediment contained sufficient polyaromatic hydrocarbon (PAH) fractions to cause induction of liver mixed function oxygenase activity (MFO), measured as ethoxyresorufin-o-deethylase activity (EROD) and bile metabolite accumulation in laboratory and field caged fish in the Biobio River (24, 25). Furthermore, four pulp mills located in the central part of the Biobio River basin were suspected to be responsible for elevated PCDD/PCDFs, due to the industrial use of elemental chlorine during the bleaching process (5). However, the low levels of PCDD/PCDFs detected in water in the pulp mill discharge areas (TEQs: 0.04 and 2.83, respectively; 5) were unable to explain the detected induction of liver EROD activity, in different wild fish species.

**Figure 3:**
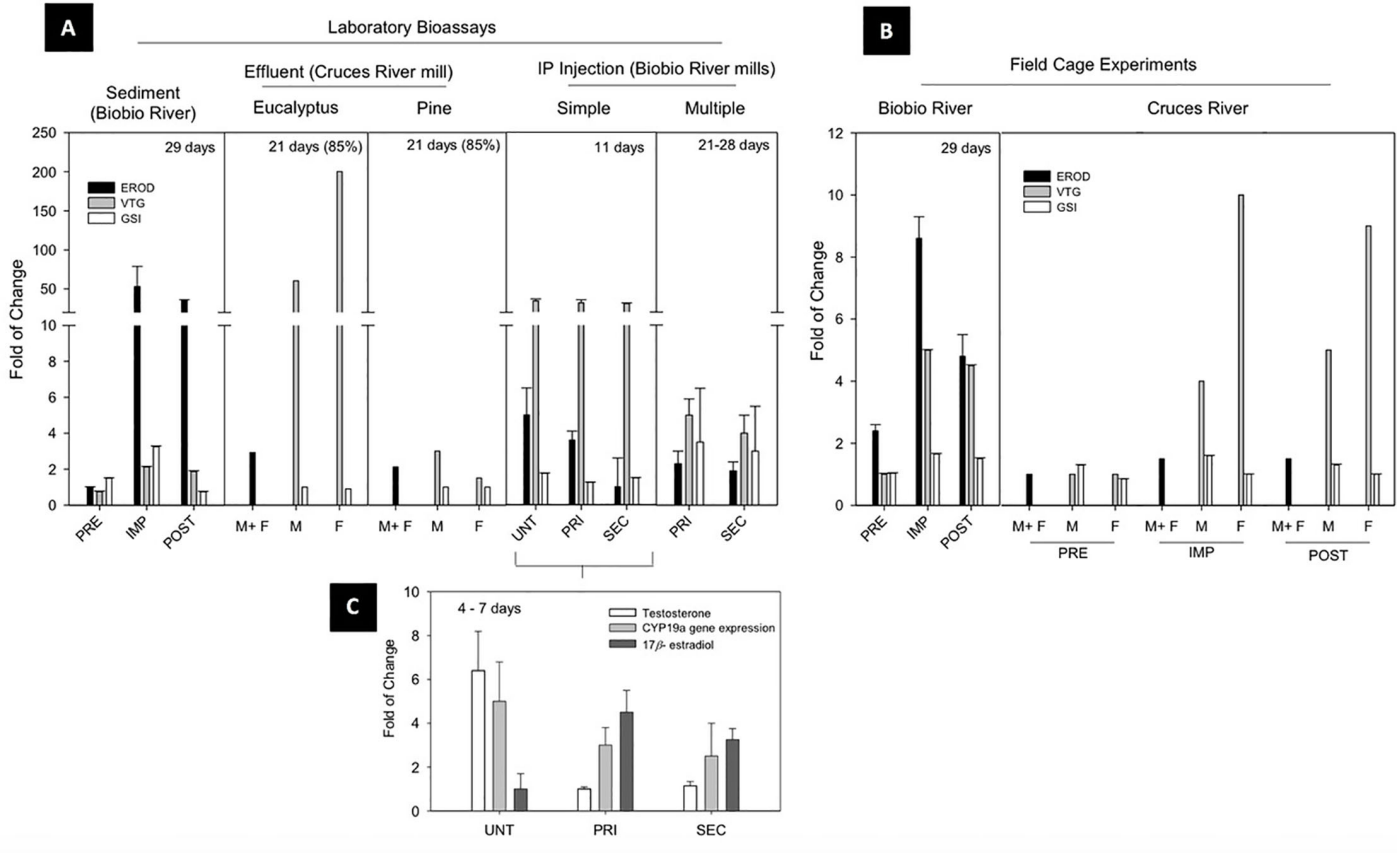
Reproductive endocrine disruption endpoints in non-native fish: Pulp and paper mill effluents evidence. **(A)** Laboratory Bioassays, **(B)** Field Cage Experiments and **(C)** Effect of mill extractives IP injection over steroids levels and *cyp*19a gene expression. PRE, preimpact zone; IMP, impact zone; POST,postimpact zone; M, male; F, female; UNT, untreated effluent; PRI, primary treated effluent; SEC, secondary treated effluent; IP, intraperitoneal; EROD, ethoxyresorufin-O-deethylase; VTG, vitellogenin; GSI, gonadosomatic index.

Despite primary and secondary effluent treatment systems implemented by most mills, a series of studies pointed to wood-derived intermediate products that could be formed and released during the normal mill operations including chlorocimenes, resin acids, chloroethenes, flavonoids, and phytoestrogens (26, 27). Some of these compounds were classified as EDCs and were suspected to accumulate in sediments, potentially becoming bioavailable (28). For this reason, our first approach was based on the evaluation of the potential endocrine disrupting effects of sediments impacted by pulp and paper mill discharges on a biological fish model under laboratory conditions (29). Hatchery reared juvenile (121 ± 14 g) rainbow trout (*Oncorhynchus mykiss*) were exposed to sediment samples obtained in an upstream reference zone (pre-impact), a zone directly influenced by pulp mill discharge (impact) and a river outlet zone (post-impact) (**Figure 3A**—sediment). High induction of liver EROD activity (53- to 36-fold of control, respectively) was found in fish exposed to the impact and post-impact zone sediment. A two-fold increase in plasma vitellogenin (VTG) was also observed. Interestingly, a three-fold increase of gonadosomatic indices (GSI) was observed in these juvenile “sexually immature” female fish exposed to impact zone sediments, showing high percentage of oocytes in a vitellogenic state. These results were the first evidence of adverse reproductive effects of pulp mill effluents evaluated under laboratory conditions in Chile.

In order to provide evidence under environmental conditions, a caged fish *in situ* exposure experiment was conducted following the same gradient in the pulp mill effluent discharge areas (20) (**Figure 3B**—Biobio River). Similar response patterns were observed, as significant increases in EROD and VTG levels of rainbow trout caged in the impact and post impact areas after a 29 d exposure. This increase in plasma VTG (4-fold of control), was again coupled with an increase in gonad maturity in even younger juvenile female fish (40 ± 13 g), provided clear evidence of persistent endocrine disrupting effects associated with pulp mill discharges.

A series of subsequent experiments were carried out examining causative agents and genetic-molecular level responses to these effluents. Untreated, primary, and secondary treated pulp mill effluents underwent solid phase extraction (SPE technique by reverse phase C-18 cartridge) to analyze their chemical composition by gas chromatography/mass spectrometry (GC-MS) (30). An intraperitoneal injection of reconstituted SPE-extracts in juvenile triploid rainbow trout demonstrated a decrease of liver EROD activity, but a 20-fold increase in plasma VTG levels (**Figure 3A**—IP injection Single). The magnitude of this consistent estrogenic effect was related to a series of potential candidate polar compounds including terpenes, abietic acids, and phytosterols. Variability in responses depended on factors such as wood feedstocks, pulping and bleaching processes, and treatment effectiveness. Interestingly, all injected extracts from the three effluents were able to increase the amount of endogenous estrogens (17β -estradiol) by induction of the *cyp19a* gene that regulates the synthesis of the enzyme responsible for converting androgens to estrogens *via* aromatization (**Figure 3C**). This estrogenic response occurred despite the presence of androgenic compounds in pulp mill effluents (31). A multiple IP injection bioassay (**Figure 3A**—IP Injection Multiple) using sexually immature female and male rainbow trout (31), demonstrated VTG induction (two to four-fold) in both sexes exposed to primary and secondary treated effluents extracts, but also indicated a potential indirect anti-estrogenic effect due to the presence of dehydroabietic acid in both of these final effluents (32).

The implementation of tertiary effluent treatment to new mills provided an opportunity to apply the integrated laboratory and field approach to assess the endocrine activity of pulp mills constructed on the Cruces River. Chiang et al. (33), showed no differences in liver EROD activity between sexes, zones, and time of effluent exposure (**Figure 3B**—Cruces River), despite significant increases in circulating VTG observed in male and female fish at impact and post-impact sites. Although no significant differences in GSI were detected, some significant increases in the frequency of maturing oocytes were observed in sexually immature females (95 ± 11 g) from impact and post impact sites, in addition to the presence of the intersex condition (eggs in testes) in male gonads.

In the laboratory, an independent waterborne exposure was conducted to different concentrations of tertiary treated effluent from a mill utilizing mixed eucalyptus and pine feedstocks (**Figure 3A**—Effluent: eucalyptus/pine). The exposure to eucalyptus effluent significantly increased liver EROD activity (two-fold control) and VTG levels (50–200 fold). For the pine effluent exposure, no significant increase of VTG was detected in females, contrary to the three-fold increase detected in exposed males. No significant differences in GSI were detected, but an increase in the frequency of maturing oocytes were observed in the immature females exposed in impact and post impact sites. Similar to the field experiment, some of the male fish showed intersex, including one male exposed to pine effluent, two males exposed to eucalyptus effluent and one male in the impact site after 11 d.

Collectively, these semi-controlled field and laboratory experiments clearly showed the potential for chemicals from pulp mill effluents to cause changes in reproductive development (abnormal increase in GSI but also its stage of female gonad maturation), biochemical markers (increase in VTG levels) and the intersex condition. It also demonstrated that these compounds were still present after modernization and advanced effluent treatments. The effluent SPE-extraction as well as the IP-Injection doses methodology, established as effluents equivalents based in the major component concentration detected in the extracts (beta-sistosterol as reference) allowed investigations of the mechanisms of toxic action and indications of the causative chemicals.

## Evidence of Urban Wastewater Effluents Impact on Non-Native Fish

The same tools successfully developed to assess Endocrine Disruption (ED) in non-native fish exposed to pulp mill effluents, were applied to assess ED effects of wastewater treatment plant effluents (WWTPs) on fish.

The Chilean water industry is a special case in Latin-America where most of the urban population have access to both clean water and sewerage services. During the early 1990s, Chile widely initiated the process of constructing WWTPs with activated sludge treatment systems. However, in urban areas, the collection of wastewater and adequate treatment covered only 20.9% of population by the year 2000 (34). With reforms conducted to address this issue (35), the proportions were improved to 96.8% and 99.8% of wastewater collection and wastewater treatment, respectively by 2016 year (36).

The implementation of WWTPs has helped to reduce eutrophication of aquatic ecosystems. However, WWTPs are generally not specifically designed for abatement of a broad range of substances, including EDCs. Substances detected in WWTP effluents include pharmaceuticals and personal care products (PPCPs) (37), as well as household, industrial chemicals and natural or synthetic hormones (38–40). In addition, the physical properties of these chemicals (i.e. solubility, volatility, adsorbability, absorbability, biodegradability, polarity, and stability) are highly variable (41, 42), affecting their behavior during treatment and consequently their removal efficiencies. Several studies have reported that many of these substances are only selectively or partially removed during wastewater treatment processes (43–48), and are discharged into the natural aquatic systems.

It is well-established that WWTP effluents can contain estrogenic contaminants at high enough concentrations to induce VTG biosynthesis, the precursor of egg yolk, in male fish (49–52), the appearance of female characteristics in male tissue (49), the reduction of gamete production and fertilization capability of male fish (53, 54), and the occurrence of the intersex conditions in male fish and altered levels of sex steroid hormones (51, 52, 55). Pharmaceutical residues have also led to a significant decrease in embryo production (56), and have also been associated with increased stress behavior (57, 58).

Currently, 47 WWTPs operate in the Biobío region, and 23 of those depositing the effluents into the Biobío River watershed, while 4 discharge directly into the Biobío River (34). A first approach to estimate the concentration of estrogens released by WWTP effluents was performed using a predictive model (59) which showed that for the Biobío River basin the equivalent concentrations of estradiol ranged between 0.01 to 1.03 ng/L (60). These concentrations were considered insufficient to cause endocrine effects on fish (61, 62). However, estimated results needed to be interpreted cautiously as the study did not consider the adsorptive properties and spatial distribution patterns of estrogens in the system.

During 2010, studies in the laboratory and the field were conducted to assess the effects of WWTP effluents on rainbow trout (63). Laboratory bioassays were carried out with juvenile rainbow trout exposed to WWTP effluents from two cites located in the Biobío basin (Los Angeles and Santa Barbara) with an approximate population of 180,000 and 14,000 inhabitants, respectively. In parallel, field *in situ* (caged) experiments were conducted at both locations. Cages were deployed in areas upstream (pre-impact), discharge (impact) and downstream (post impact) of WWTPs located in the Santa Barbara and Los Angeles (Quilque stream) cities.

**Figure 4:**
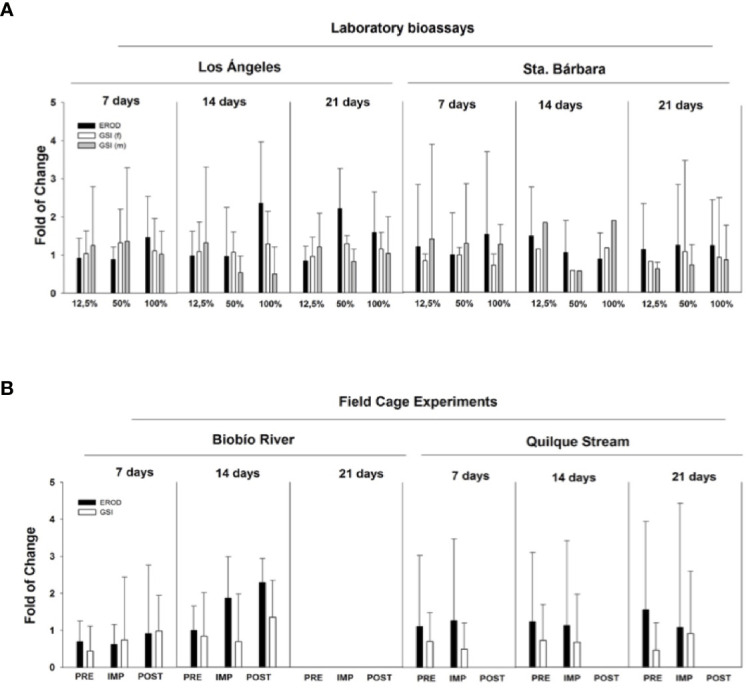
Laboratory bioassays and field cage experiments with *Oncorhynchus mykiss* exposed to wastewater treatment plant effluents located in Biobío River basin. **(A)** Laboratory bioassay gonadosomatic index (GSI) and ethoxyresorufin-O-deethylase (EROD) in females (f) and males (m) exposed to 12.5%, 50%, and 100% effluent for 7, 14, and 21 days. **(B)** caging experiments show GSI and EROD endpoints after the exposure in river (PRE, pre-impact zone, IMP, impact zone, POST, post-impact).

There were no clear alterations in gonadal size (GSI) in fish exposed to WWTP effluents. GSI increased at higher concentrations (50% and 100% of effluent) as exposure times increased (**Figure 4A**) in male (2.5-fold) and female (2-fold) rainbow trout for both effluents. Rainbow trout for caging studies were immature, so males and females were pooled together. GSI increased slightly (0.5 fold) at the impact site at Santa Bárbara after 14 days of exposure and at the impact site at Los Angeles (1.0 fold) after 21 d of exposure (**Figure 4B**). However, no significant changes were report in any all the experiments. Not all fish were available at both sites as vandalism resulted in lost fish.

The biomarker detoxification enzyme EROD was also used to assess the exposure of WWTP effluents on rainbow trout. In general, EROD activity showed an increased response when effluents from Los Angeles were more concentrated and times of exposure were increased in the laboratory. Effluents collected from Santa Barbara did not show a clear pattern in EROD activity (**Figure 4A**). In the field experiment, the most relevant results are those related to EROD activity, showing an increased response at the impact site and post-impact (2.5 fold) after 14 d exposure to Santa Bárbara WWTP effluents. For WWTP effluents belonging to Los Angeles, EROD activity did not show significant spatial or temporal changes (**Figure 4B**).

**Figure 5:**
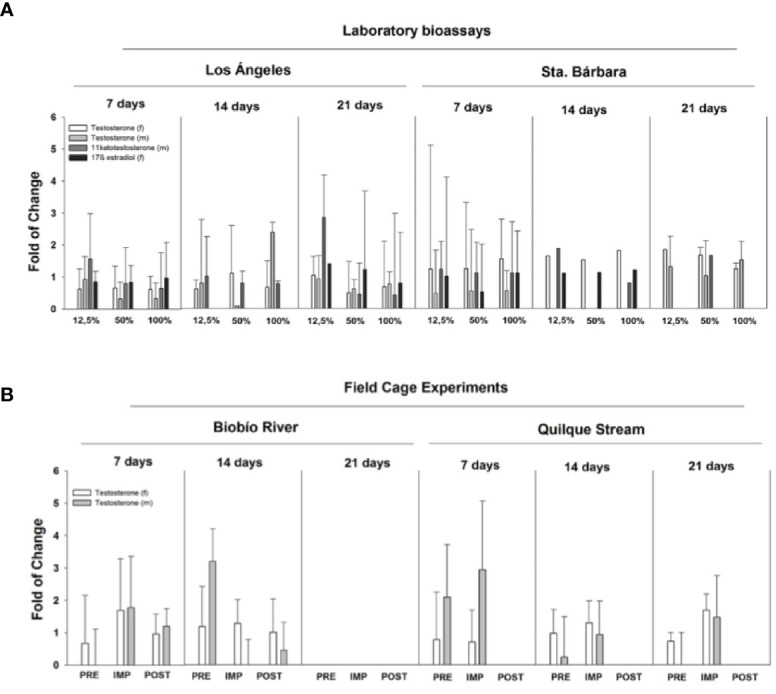
Steroidal hormone levels in male (m) and female (f) rainbow trout exposed to WWTP effluents in the Biobío River basin. **(A)** Laboratory bioassays show responses in testosterone, 17β-estradiol, and 11 ketostestosterone to 12.5%, 50%, and 100% effluent exposure after 7, 14, and 21 days. **(B)** field cage experiments are reported for PRE, pre-impact zone, IMP, impact zone, POST,: post-impact zone of Biobio river basin.

Steroid hormone levels were measured in fish from both the laboratory and field caging studies. **Figure 5A** shows laboratory exposures to Los Angeles City WWTP effluent, with circulating hormones in female plasma (17β-estradiol and testosterone) increased relative to controls. Generally both female and male fish, exposed to effluent increased steroid levels in the plasma. For example, females 17β-estradiol increased in all treatments relative to control, but it was not significant. Males exposed to the same effluents (bioassay Los Angeles) concentration, showed similar responses as females with increases in gonadal hormones (testosterone and 11 ketotestosterone; **Figure 5A**). This hormonal behavior is mainly related with maturity and the spermatogenesis process. Only testosterone was measured in the field cage experiment. Female testosterone increased after 7 d exposure in Santa Barbara MWWTP effluent impact site (Biobío River) and in Los Angeles WWTP effluent (Quilque stream) the increase was at 21 d in the impact site. On the other hand, male testosterone increased significantly at Los Angeles impact site at 7 d of exposure and no significant changes were observed at the Santa Barbara location (**Figure 5B**).

Both WWTP effluents showed different effects, probably due to the differential features of the population living in both towns: Los Angeles is a much larger town than Santa Barbara. However, these results must be taken with caution since no statistically significant differences were found, and the effects were not as severe than those reported to as *Evidence of Pulp Mill Effluent Impacts on Non-Native Fish.*

A summary of all lab bioassays and the field experiments with PPMEs and WWTP in rainbow trout are provided in **Table 1**.

**Table 1:** Most relevant biological responses to EDCs in rainbow trout at Chile.

River	Type of stressor	Type of experiment	Reproductive endpoints	Other parameters	References
Cruces River basin	Pulp and paper mil effluent (ECF with tertiary treatment)	Bioassay	++VTG protein in blood of juvenile m & f (Eucalyptus ECF effluent) +VTG protein in blood of juvenile m & f (Pine ECF effluent) + gonads maturation in juvenile f & m. + intersex in m gonads		Chiang et al. (33)
		Fish caging	++VTG protein in blood of juvenile m & + gonads maturation in juvenile f & m. + intersex in m gonads		
Bio Bio River Basin					
	Pulp mil effluents (BKME)	caging	++ VTG-protein in blood + gonad maturation in f + GSI in f	+ EROD activity + LSI	Orrego et al. (5, 20)
	Pulp mil effluents (BKME)	Lab bioassay	++VTG-proteins in blood + gonad maturation in f + GSI in f + 17βestradiol in tp	+ EROD activity + LSI + LDH/CS activity + hepatic CYP19arom gene expression in tp	Orrego et al. (20, 30, 31)
	Sewage treatment plant effluent	Lab bioassay	+GSI in females and males + testosterone in females and males	+ EROD activity	Saavedra (63)
	Sewage treatment plant effluent	caging	+ testoterone in females and males	+ EROD activity	Saavedra (63)

BKME, bleached Kraft Milll effluent; TMP, thermo-mechanical pulp; ECF, elemental chlorine free; GSI, Gonadsomatic Index; LSI, Liversomatic Index; K, condition factor; VTG, vitellogenin; LDH, lactate dehydrogenase; CS, citrate synthase; m, male; f, female; tp, triploids; +, increase; −, decrease.

## Evidence of Endocrine Disruption in Fish Native to Chile

Chilean native freshwater fauna have been poorly studied in terms of the stress caused by industrial effluents. This presents a problem for conservation, as the greatest richness of freshwater species occurs in the Central-South zone of the Chilean province, the area where the greatest anthropogenic pressures are concentrated. The use and extraction of water may impact abundance and distribution of native fish in highly disturbed systems (11). Ecotoxicological tools complement the traditional estimates of environmental contamination, such as measurements of chemical residues in water, sediment or biological samples, and help to link the observed effects to their possible cause, generating data that can help in the mitigation of impacts and the conservation of aquatic biota.

Only a handful of species examined to date have characteristics sought as an indicator species in freshwater systems of central Chile. *Percilia gillissi* (and its congeneric specie *P. irwini*, restricted to the Biobío River basin) and *Trichomycterus areolatus* meet the requirements within our study strategy. *T. areolatus* has a wide abundance and geographic distribution, being reported from the Limari River Basin to Chiloé (64). Although, it is considered a “vulnerable” species, with the abundance and distribution data of these authors, a great plasticity in the use of habitat and trophic niche is evidenced, adapting to the favorable conditions of the environment. Similarly, the reproductive data provided by Manriquez et al. (65), Habit et al. (11) and Chiang et al. (66, 67) coincide, indicating the spawning season (spring to early summer). *T. areolatus* has a benthic and benthophagous life strategy, with a closer contact with the sediment, an environmental matrix widely described as the main reservoir of lipophilic compounds. Orrego et al. (29) established that the sediments in the Biobío river are the main reservoir of the compounds responsible for the changes reported at the molecular, biochemical, and organismic level in laboratory experiences with *O. mykiss* and which they associated with the discharge of cellulose effluents.

*P. gillissi* has a distribution between Aconcagua and Puerto Montt (64). It is found preferentially in rithron environments and swimming at mid-water, its diet is mainly benthophagus as reported by various authors. Chiang et al. (66, 67) showed that this species shares the spawning season with *T. areolatus*, and overlapping with its congeneneric species (*P. irwini*). *Percilia gillissi* is considered “vulnerable” between the Biobío and Los Lagos regions, due to decrease in abundance and habitat alteration according to Habit (pers. Comm.), while *P. irwini* is listed as endangered by the same authors.

For Chile, the effects of EDCs have been mainly studied in the Ñuble and Biobío regions (see **Figure 1**) on the Itata and Biobio river basins as pulp and paper production and agribusiness effects have been observed in the local aquatic fauna (12, 32, 66, 67, **Table 2**). If we focus on the responses of native fish, at the community level, a decrease in diversity (H´), species richness (S) and abundance have been observed in a longitudinal gradient of the Biobío River, contrary to the normal patterns known for rivers not influenced by these industries (11). There is a change in the structure of fish assemblages, with an increase in the distribution range and abundance of exotic tolerant species in the Biobío River (11). At the Chillan river basin, Orrego et al. (69) reported an increase in the EROD response in *Trichomycterus areolatus* in an upstream-dowstream gradient, as sewage discharges (treated and untreated) increase, as well as runoff of agricultural and forested areas.

**Table 2:** Most relevant biological responses to EDCs and other stressors in native fish from Chile.

Zone/species	Type of stressor	Reproductive endpoints	Population and Community responses	Other parameters	References
Biobio River basin Multiple fish species	Various Pulp mil effluents (BKME, TMP, ECF), various STPE and runoff from agricultural-forestry activities		- abundance (CPUE) native species; - Diversity & richness of native species; + abundance & distribution range of tolerant exotic species		Habit et al. (11)
*P. irwini*	STWPE (treated) and Pulp and paper mil effluent (ECF)	+ VTG-like-phospholipoproetins (mucus); - testosterone m + 17β -estradiol in f (short term); − 17β -estradiol in f (long term)			Bahamonde et al. (68)
Chillan River Basin Multiple fish species	Untreated sewage discharge, runoff from agricultural-forestry activities		- abundance (CPUE) native species; + abundance tolerant exotic species		Orrego et al. (69)
*T. areolatus*	Untreated sewage discharge, runoff from agricultural-forestry activities			= AchE activity, + EROD activity along river gradient (Epirithron<Metarithron<Hiporithron)	
*T. areolatus*	Untreated and treated sewage discharge, runoff from agricultural-forestry activities	+ estrogenic activity in m		+ skull fluctuating asymmetry	Bertin et al. (70)
Itata River Basin *P. gillissi*	Pulp mil effluents (BKME)	+ 17β -estradiol in f; - 11ketotestosterone in m; + GSI; Disruption in oocyte production	- abundance of juvenile fish, smaller adult fish	++ EROD activity + LSI	Chiang et al. (67)
*T. areolatus*	Pulp mill effluents (BKME)	+ 17β -estradiol in f + GSI	- abundance of juvenile fish, smaller adult fish	+ EROD activity + LSI	Chiang et al. (67)
Itata river outlet, Cobquecura Bay, Coliumo Bay *P. adspersus*	Treated and untreated sewage discharge, runoff from agricultural-forestry activities. Pulp mil effluents (BKME)	+ VTG in m; + frequency in early stages of spermatogenesis in m; +GSI in m			Leonardi et al. (71)
Choapa River Basin *T. areolatus*	Metals, oxidative stress, and agrochemicals	− hepatic gene expression (VTG and Erα) in f; − ovarian gene expression (HSP and Erα) in f		+ hepatic gene expression (CYP1A and HSP70) in m - hepatic gene expression (HSP and AHR); + gill metallothionein gene expression in f	Ali et al. (72)

BKME, bleached Kraft Milll effluent; TMP, thermo-mechanical pulp; ECF, elemental chlorine free; GSI, Gonadsomatic Index; LSI, Liversomatic Index; K, condition factor; VTG, vitellogenin; m, male; f, female. +, increase; −, decrease.

**Figure 6:**
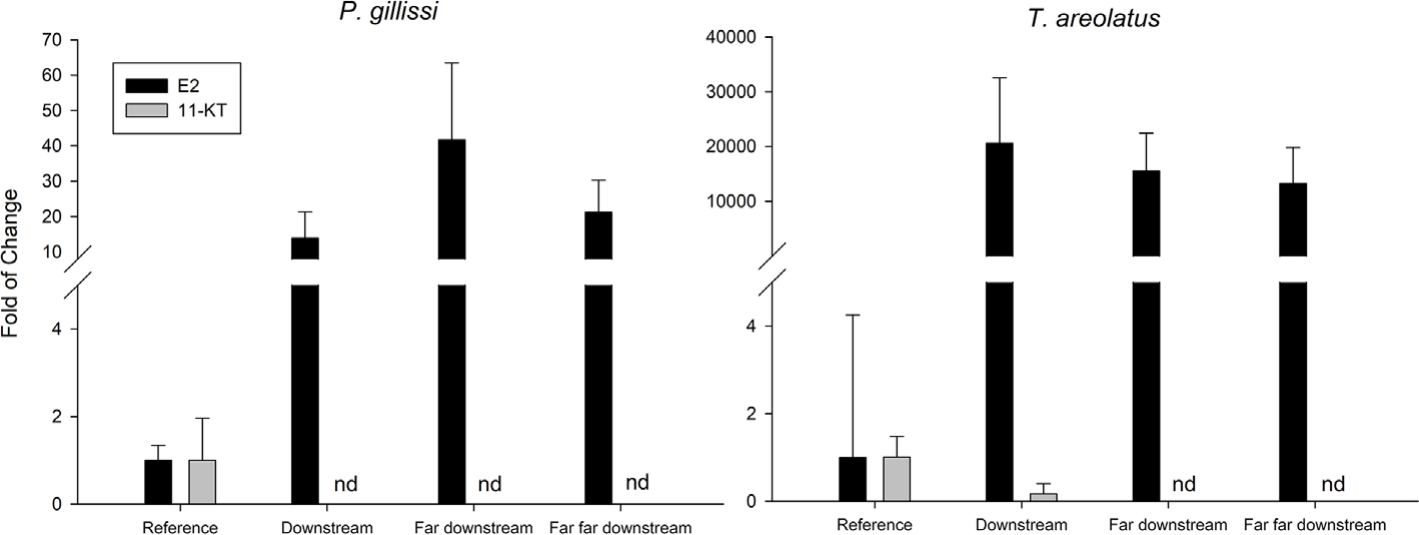
Steroidal hormones 17β-estradiol (E2) and 11k-testosterone (11-KT) in native fish in a river effected by PPME discharge. (nd, non detected).

**Figure 7:**
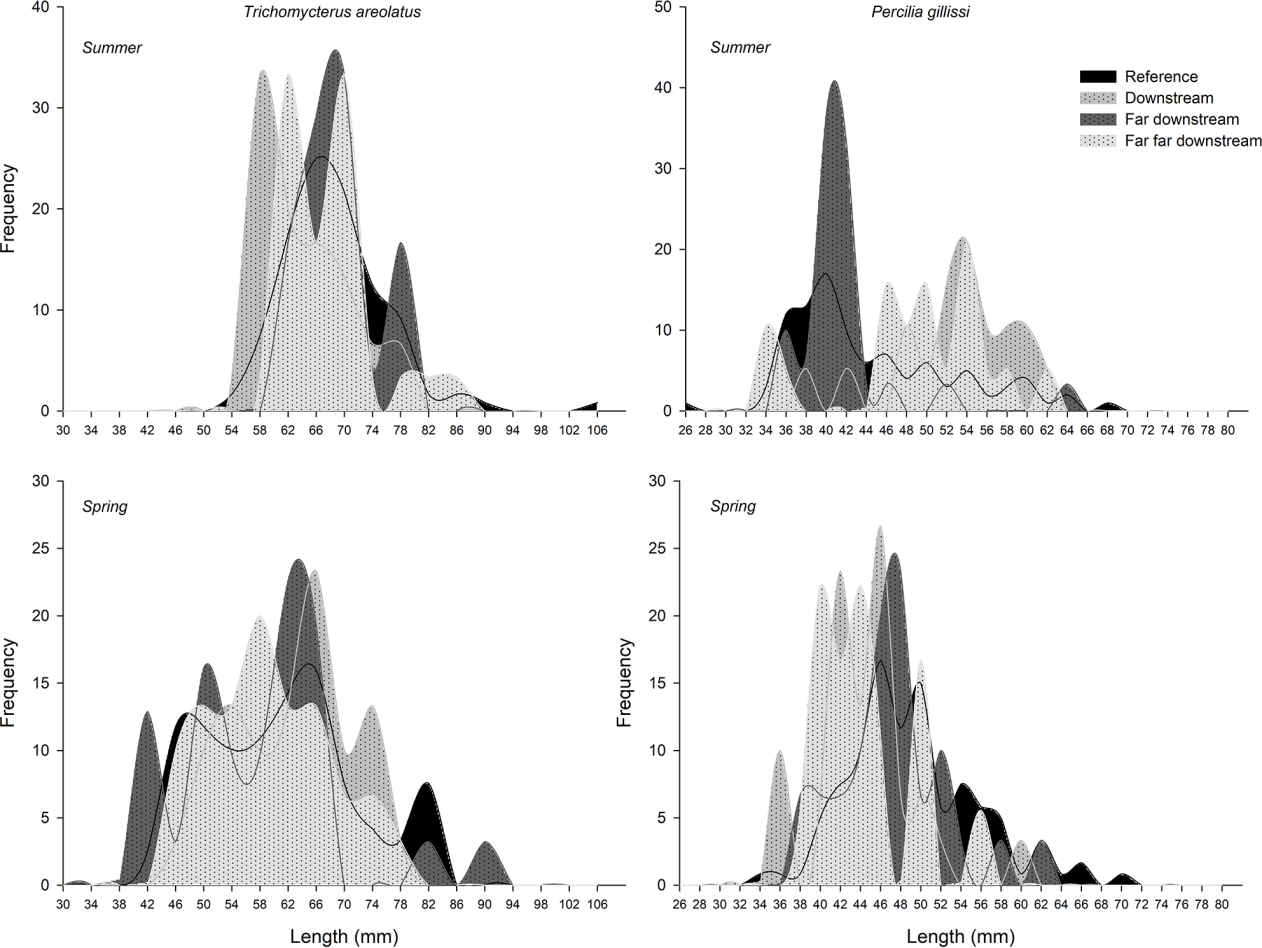
Changes in the fish size frequency between upstream and downstream Elemental Chlorine Free-PPME discharge sites.

The first evidence of ED in wild fish native to Chile was observed in the study by Chiang et al. (67). This study showed evidence of reproductive disruption at different levels of biological organization, suborganismal responses, and additional population level effects in *Percilia gillissi* and *T. areolatus* exposed to pulp mill effluents in the Itata River. A clear estrogenic effect was observed downstream of the effluent discharge, with an induction of gonadal production of 17β-estradiol (**Figure 6**) and a higher frequency of oocytes in advanced stages of maturation, with a consequent increase in the gonadal size of the females. Different responses were observed in males, with a decrease in the production of the main androgen in fish, 11-ketotestosterone (**Figure 6**) and slight increases in the size of the gonad. Increases and decreases in the relative size of the liver and condition observed, which together with an induction (greater in *P. gillissi*) of EROD activity downstream of the discharge indicate metabolic disruption. At the population level (**Figure 7**), a loss of individuals of greater size and juveniles was observed, which could be linked to the reproductive and metabolic aspects observed, but require further study.

An estrogenic response was observed in the only study of ED in marine coastal fish in Chile. An induction of VTG in male *Paralichthys adspersus* on the coast of the Biobío region, as well as a disruption of the maturity cycle of the testes, with an increase in the frequency of early stages of spermatogenesis, followed by an increase in the GSI of these fish at the mouth of the Itata river which receives pulp mill effluent discharges, wastewater (treated and untreated) and agroforestry runoff (71). Evidence of estrogenic effects in wastewaters from these basins triggered the effort to develop non-lethal EDC assessment tools in native fish, given their high conservation value. Thus, Bahamonde et al. (68) demonstrated an increase in VTG-type phospholipoproteins (Vtg-like-phosphoproteins) in mucus on *Percilia irwini* exposed to wastewater from the Biobío River basin. Fish exposed to cellulose effluents as well as treated wastewater also showed an increase in these Vtg-like-phosphoproteins, similar to fish exposed to EE2 (10 ng/g). In parallel, a decrease in testosterone production in males and an increase in 17β-estradiol in females were observed in the first half (7 d) of the bioassay experiment, and then declined (12 d).

In the north of Chile, in the Choapa river basin, responses at the molecular level have shown effects of ED in areas of the river with multiple stressors, such as metals and agrochemicals, with a decrease in hepatic and ovarian gene expression of *erα*, as well as decreased hepatic *vtg* and hepatic *hsp* gene expression in *T. areolatus* females (72). These same authors showed an increase in hepatic gene expression of *cyp1a* and *hsp70* in males, decrease in hepatic gene expression in *ahr* in liver and increase of metallothioneins in females. Although most of the few studies of ED in native wild fish in Chile have focused on reproductive aspects (addressing metabolic aspects of growth), we cannot rule out other effects of these compounds on fish ontogeny. Bertin et al. (70) showed a high estrogenic activity in sediments and males of *T. areolatus* from the Chillan river basin, in areas of high agroforestry use and discharge of treated and untreated wastewater, which may be associated with fluctuating asymmetry effects of skulls of this same species, and there is evidence of effects on the development of fish in this basin. In addition, intersex condition have been found on *P. gillisi* habiting the same watershed (Bahamonde et al., in prep).

A summary of the findings of endocrine impacts in native fish are summarized in **Table 2**.

## Discussion

The evidence shown in this review demonstrates the complexity of addressing multiple stressors and complex mixtures across a range of Chilean aquatic ecosystems. In the case of ED, an integrated approach as discussed by Barra et al. (73) is required, where both chemical and biological tools may be used to demonstrate effects on the sentinel species but also to link those effects to sources and causative chemical(s) compounds.

An interesting issue raised by our analysis is the difference observed between different sources of EDCs (industrial and urban wastewaters) in the responses in the organisms tested in Chile, in particular for the non-native fish. Research on the impact of Chilean pulp mill effluents indicates an overall estrogenic effect and a negative impact on reproductive function of exposed non-native fish (31), which is consistent with recent results obtained in bioassays carried out with effluents from other pulp producing countries such as Brazil, New Zealand, and Canada (74, 75). Results included induced activity of the CYP19arom enzyme, responsible for the transformation of androgenic compounds into estrogens through aromatization (31), exerting a final estrogenic effect in non-native fish. Correspondingly, increased levels of endogenous plasma 17β-estradiol were found in fish chronically exposed to 100% of a Chilean WWTP effluent after 21 d (63). Numerous emerging contaminants such as pharmaceuticals used in human and veterinary medicine, including nonsteroidal anti-inflammatory drugs, painkillers, antibiotics, lipid regulators, steroid hormones, and fungicides, have been detected in high concentrations in the Chilean aquatic environments (66, 76). These chemicals and degradation products within these mixtures may be responsible for the increase of EROD activity, altered steroidal hormones, and reproductive disruption such as the increased degree of gonadal maturation.

Similar results have been described for urban effluents in Africa, where an effects-directed bioassay in Zimbabwe detected estrogenic effects by analyzing similar endpoints to our studies in native fish (77). Similar findings in China and other Asian countries also reveal that the proper use of transcriptomic techniques could approach the identification of the causative chemicals of the endocrine effects revealed by native fish exposed to urban effluents (78). The need to strengthen the integrated analysis for EDCs in developing regions is clear, which underscores this as a global issue.

The results of <15 years of study in Chilean ecosystems indicate that despite the differences in species and complexity of the chemical composition, fish respond through similar mechanisms to elicit responses at higher levels of biological organization (e.g. populations). Disruption of endocrine function may cause a cascade of responses that lead to adverse outcomes in fish like changes in fish populations or communities, as has been evidenced by Kidd et al. (79). The complete shutdown of 11-ketotestosterone in males of *P. gillissi* and *T. areolatus*, plus the evidence of early maturation in juvenile rainbow trout (and intersex in male gonads) exposed to PPMEs could be leading to a similar scenario, with a lack of reproductive capacities to maintain sub-populations in the sites downstream the PPMEs discharge (67). Length-frequency data from Chiang et al. (67) showed that the subpopulations of native freshwater fish were limited to intermediate sizes, with no larger adults, juveniles or YOY (young-of-year). In this case, the Itata River, being an open system (contrary to the research conducted by Kidd et al. (79) at the Experimental Lakes Area) could be subsidized by individuals recruited from downstream or upstream that could be colonizing those areas and are exposed in a secondary way to the effluents. Still, this remains a hypothesis and needs further research.

There are shortcomings for the study of EDCs in Chile, especially concerning native freshwater fish. It is necessary to understand that fish are constantly subjected to environmental stress (21, 22) before planning EDC effect studies, including changes in temperature, hypoxia, sediment loads, flow rate, among others. To understand and to have the ability to extrapolate the effects observed in the individual organism with a chemical or mixture of chemicals having endocrine disruptive properties, cellular and physiological seasonal variations associated with changes in metabolic rates and fish reproductive status must be taken into consideration when evaluating the impacts on individuals and populations (9, 21, 33, 66–78, 80).

The selection and interpretation of biological endpoints must consider the characteristics of the life history, the evaluation of the seasonal variability of reproductive parameters (sex hormones, oocyte development, GSI, size structure), metabolic responses (condition factor, LSI) and other biomarkers of fish health which permit us to define: (i) what feature of the entity is affected, (ii) the degree of modification of this feature, and (iii) the potential degree of involvement of EDCs. There is therefore a need to analyze in more detail the history of life and reproductive behavior of these native species (9).

As already mentioned, for Chile there are few studies that detail reproductive aspects of freshwater fish beyond the spawning season (66, 67, 78, 80). The paucity of such an understanding is a major deficiency when evaluating the effects of EDCs. Furthermore, only the studies by Chiang et al. (67) have reported the seasonal variability of sex hormones in freshwater fish for Chile and only in two species (*P. gillissi* and *T. areolatus*), a crucial factor in understanding and evaluating the effects of EDCs on wild populations exposed to these pollutants. These data, together with the analysis of other reproductive parameters at different levels of biological organization (organ, individual, population) will allow us to accurately assess the sustainability of wild fish populations that are exposed to EDCs. This is how the analysis of sexual hormones and histology in *T. areolatus* allowed the results of Manriquez et al. (65) in making macroscopic observations of the female gonad to be updated.

The same analysis facilitated a description of gonadal development of *P. gillissi* for the first time. Seasonal histological analysis of male and female gonads (80), together with the analysis of 11-ketotestosterone and 17β-estradiol hormones (67), allowed descriptions of these species as gonochorists, with asynchronous gonadal development (among spring-summer), providing a solid basis of comparison to assess the reproductive effects of EDCs. Importantly, Bahamonde et al. (81) carried out an in-depth analysis of the intersex in various wild fish species, given that the occurrence of intersex is a direct effect of exposure to pollutants with EDC action. This factor is relevant when evaluating EDCs, given that the same authors highlight in their review that intersex has a natural incidence rate between 0.5 to 55% of the specimens, depending on the species, and may confuse the study design. In this regard, Arratia and Quezada-Romegialli (82) comment on the development of *P. gillissi* gonads, indicating that this species presents a prostatic hermaphroditism, based on conversations and observations by Riffo during the years 1974–1975 (G. Arratia comm pers). However, Riffo (83) indicates that the development in all tests (1 male out of 4 in August 1974; 1 male out of 22 in January 1975) is a rudimentary non-functional hermaphroditism that he attributes to an involution process. Bahamonde et al. (81) point out that intersex can occur, as we already mentioned, spontaneously in gonochoristic species exposed to EDCs. This fact together with the evidence of Chiang et al. (67, 80) who did not observe this phenomenon and could determine the size of first sexual maturation for both sexes separately, suggests that the specimens found by Riffo (83) in the “Peuco” stream (southern limit of the province of Santiago, an area of high agricultural use and pesticides with potential ED) are the first evidence of EDCs in Chile.

Thus, the lack of knowledge of the basic biology and life history of Chilean fish, especially related to sexual development, along with habitat and landscape characteristics, are risk factors for their conservation. This factor along with EDCs threat, have been neglected by aquatic ecologists in Chile, despite the fact that pollution is one of the five greatest threats to the diversity of freshwater systems (16, 17). Similarly, the conservation status of most freshwater fish in Chile is poor, vulnerable or endangered, so the development of non-invasive/non-lethal tools for the effects of EDCs, such as VTG-like-phospholipoproteins in mucus (68) are an ideal approach.

Besides the restrictions given in the above paragraphs, there is a need to establish a more robust cause-effect relationship for correctly interpreting endocrine responses towards chemical pollution from other potential confounding factors. We therefore recommend to address these issues with a battery of approaches including non-lethal tools, that needs to be validated, toxic “omics” for analyzing mechanistic effects, but related to more ecologically relevant endpoints such as populational effects. These strategies must be complemented with more sophisticated sampling tools such as passive samplers and instrumental analysis tools such as non-targeted analysis of contaminants (73).

One of the lessons learned in this journey is that standardized protocols must be established for allowing a robust effects-based biomonitoring program. Without basic information about the reproductive performance of the fish analyzed, it is risky to conclude impacts of EDCs. Therefore a basic knowledge of the reproductive behavior of the species used as bioindicators is a requirement for a successfully implemented project. In addition, biomarkers could be added for a mechanistic understanding of the reproductive effects, a very useful marker is the VTG protein and hormonal levels. Both indicators are very useful insights for observing EDCs impacts.

Water quality monitoring in Chilean rivers needs to be improved. Very few rivers have water quality regulations and usually, the number of parameters measured is very limited. For example, the Biobio River water quality regulation enacted in 2015 only controls 15 parameters which are measured only four times per year. Therefore an effects assessment strategy is needed if we want to conserve the fish freshwater biodiversity (73).

egulatory improvements are also needed to effectively address the issue of EDCs in Chilean rivers, as far as the update of emission regulations by including both toxicity bioassays as well as an effects assessment procedure in the verification of impacts in the receiving environment, with the goal of verifying if the regulations are effectively protecting the rivers, as is conducted in Canada (6). Emphasis is also needed on abatement options, measuring impacts is very important, but it is highly necessary to also consider how to find technologies to mitigate the impacts as well as the regulatory measures to incentivize industry to implement them.

The introduction of multiple complex mixtures such as urban and industrial effluents in rivers of the Mediterranean zone may be one of the factors that reduces biodiversity and could also explain the homogenization observed in Chilean fish fauna in recent years (84).

Expanding the approaches underlined in this paper to other river basins in Chile is recommended to detect and address the occurrence of reproductive effects caused by anthropogenic sources on freshwater fish. This strategy should be established based on a more robust knowledge of the reproductive aspects in native species and in a clear metric of impacts, such as critical effect sizes to address the following questions in an adaptive monitoring paradigm: what should be considered an adverse impact? What difference should be considered as significant on a biological basis?

## Conclusions

It is clear that Chilean Waters are exposed to a variety of EDCs. The complexity of multiple stressors in watersheds that need to be addressed should not be a deterrent for initiating study. Organisms within the receiving environment integrate the stressors and complex situations and can be approached with the integrated approaches reviewed here, analogous to Environmental Effects Monitoring. Assessment of effects in the receiving environment is critical for supporting and designing regulation and remediation.

A tiered field-based assessment allows us to determine if there are effects, and then work toward identification of cause and remedial actions. Mechanistic studies allows for making linkages to sources. The analysis of other reproductive parameters at different levels of biological organization (organ, individual, population) will allow us to accurately assess the sustainability of wild fish populations that are exposed to EDCs.

It has been demonstrated that regardless of the type of treatment, pulp mill effluents can contain compounds capable of impacting endocrine systems. There were clear differences observed between different sources of EDCs (industrial and urban wastewaters) in the responses in the organisms tested in Chile, in particular the non-native fish. Although not directly comparable as they have a different source, PPME and WWTP effluent, seem to cause similar chronic estrogenic effects during fish waterborne exposure. There is a need for future studies on basic biology of native fish species, and an improved understanding on reproductive development and variability across Chilean systems. This last point is extremely important, as Chile´s natural habitat variability would play a role in modulating the fitness and reproductive outcome in different basins, for the same species, introducing a major risk factor for the conservation and the threat of EDCs.

## Data Availability Statement

The raw data supporting the conclusions of this article will be made available by the authors, without undue reservation.

## Author Contributions

RB participated in the outline planning, drafting, and writing. GC participated in the native fish impacts, pulp and paper mill discharges, and writing. MSa participated in the urban wastewater impacts and writing. RO participated in the pulp mill discharges impacts on introduced fish and writing. MM participated in the editing and review. MH participated in the editing and review. MSe participated in the editing and review. FT participated in the writing and review. KM participated in the editing and review. PB participated in the editing and review. All authors contributed to the article and approved the submitted version.

## Funding

The authors would like to thank the different funding sources. RB would like to especially thank ANID/FONDAP/15130015 and ANID/FONDECYT/1180063 grants, ANID – Millennium Science Initiative Program – ICN2019_015. PB is supported by Nucleo Milenio INVASAL funded by Chile’s Government Program, Iniciativa Cientifica Milenio from the Ministerio de Economia, Fomento y Turismo and FONDECYT Initiation 11180914.

## Conflict of Interest

The authors declare that the research was conducted in the absence of any commercial or financial relationships that could be construed as a potential conflict of interest.
